# Internationalization of higher medical education in the post-COVID-19 era

**DOI:** 10.1080/10872981.2023.2202459

**Published:** 2023-04-13

**Authors:** Norbert Skokauskas, Branko Aleksic, Madeleine Moe, Divya Rayamajhi, Anthony Guerrero

**Affiliations:** a Regional Centre for Children and Youth Mental Health and Child Welfare, Central Norway, IPH, Norwegian University of Science and Technology, Trondheim, Norway; b Child and Adolescent Psychiatry Section, World Psychiatric Association, Geneva, Switzerland; c Department of International Medical Education, Nagoya University Graduate School of Medicine, Nagoya, Japan; d Norwegian Embassy in Ottawa, Ottawa, Canada; e Department of Political Science, University of Oslo, Oslo, Norway; f Department of Public Health and Nursing, Norwegian University of Science and Technology, Trondheim, Norway; g Department of Psychiatry, University of Hawaii School of Medicine, Honolulu, HI, USA

**Keywords:** Internationalization, international students, curriculum, mobility, COVID-19

## Abstract

COVID-19 pandemic has caused disruption in higher medical education and healthcare worldwide. To thrive in times of uncertainty, medical higher education institutions have to adapt to the post-COVID-19 era and innovate its international activities. To make a difference in societies locally, nationally and internationally, they will have to enhance their global presence. Internationalization is the best way to the exchanging of knowledge, enhancement of the medical curriculum, and mobilization of talent and resources for research and teaching. To remain competitive, universities will need to expand their international activities. This paper highlights several suggestions to enhance internationalization of medical higher education institutions in the post-COVID-19 era.

Internationalization is the best way to the exchanging of knowledge, enhancement of the medical curriculum, and mobilization of talent and resources for research and teaching. To remain competitive, universities will need to expand their international activities. To thrive in times of uncertainty, they have to adapt to the post-COVID-19 era and innovate its international activities. To make a difference in societies locally, nationally and internationally, they will have to enhance their global presence.

Below we highlight several suggestions to enhance internationalization of medical Higher Education Institutions (HEI) in the post-COVID-19 era:

1. Universities will be able to recruit more outstanding and diverse international students by strengthening existing partnerships, working closely with their alumni, targeting carefully selected new geographic regions, and taking advantage of emerging big data analytics. *Challenges*: Physical recruitment activities suffered due to COVID-19, and some economies, hit hard by the virus, may witness a decline in numbers of outbound students. While China will remain the largest student recruitment market in the near future, its overall weak growth was evident even before pandemic [1]. *Opportunities*: There is a need and an opportunity for diversification of recruitment portfolios going beyond high-value regions and countries (i.e., China, Malaysia), with a focus on ‘potential growth regions’ such as Sub-Saharan Africa, Central Asia, and others. Decision support systems go beyond clinical practice [2] and new digital recruitment platforms are bringing big data analytics into the recruitment system [3].
2. Universities will have to provide students with diverse mobility opportunities rendering benefits for individuals and communities at a local, regional and global level. *Challenges*: Ecological problems exemplified by climate change will affect international travel. There will be a need to make digital interaction simple and natural. *Opportunities*: A positive consequence of digitisation of classes during the pandemic has been the flexibility that it has given students. This can be an opportunity to cater to the needs of students who are working, commuting or traveling.
3. Universities will have to empower staff members to be facilitators and advocates for comprehensive internationalization and create an environment and a culture to nurture creativity and achievement, with a focus on quality and excellence. There is a need to identify and invest in opportunities that promote global mobility for staff so that they become more internationally knowledgeable and interculturally skilled. *Challenges*: Some faculty members, especially in countries where the English language is not commonly spoken, may be resistant to change and comprehensive internationalization (partly because of language barrier). However, the survey by the International Association of Universities suggests just the opposite: internationalization gives a unique *opportunity* for professional, personal growth and modernization of the education process [4].
4. The internationalized curriculum will ensure that all our students are exposed to international and intercultural perspectives. Having international partnerships as part of a degree shall give more legitimacy to the program and shall make the students adapt to the globalized world. Online learning innovation may be important driving force for an internationalized curriculum; however, the curriculum should be engaging, and communication should be interactive to provide results that are similar to an in-class experience. *Challenges*: Internationalization of the curriculum (IoC) has been emphasized in many universities for a period of time, and might receive less attention from the staff in the post-COVID −19 era. However, IoC has been defined as an ongoing process and *opportunity* for the incorporation of international, intercultural and global dimensions into the content of the curriculum, as well as learning outcomes, assessment tasks, teaching methods and support services of the study programme [5].
5. The future research collaborations have to tackle important global problems outlined in UN Sustainable Development Goals (SDGs) [6]. Although climate change has been identified as one of the greatest threats to health, medical school curricula have very little coverage of its health consequences [7]. Most students are concerned about the future and wish to make environmentally conscious decisions. *Challenges*: There are limited funding opportunities for global health and related topics. *Opportunities*: Universities have to invest in young researchers to develop international excellence and to support career development that spans the postgraduate, early career, and international leadership stages. The internationalized curriculum shall cover SDGs.
6. In post COVID −19 era partnerships and networks have to bring diverse viewpoints, inspiring discussions, additional resources, and rewarding activities to address global issues. Alumni around the world has to play an important role in establishing new partnerships and networks. *Challenges*: There is growing competition among HEIs for a place in the best partnerships and networks. Partnerships will allow to embrace *opportunities* not otherwise available.
7. Universities will have to have an inclusive system for monitoring, reflecting upon, evaluating and reviewing internationalization progress. *Challenges*: There is a need to be sensitive to understandings and attitudes towards internationalization, and miscommunication between stakeholders. Financial restrictions may also pose difficulties. *Opportunities*: Such a review process will help to strengthen competence, confidence, and credibility.

Practical solutions for internationalization have to be tailored to individual HEI needs and recourses and be flexible and adaptable.
